# Supplementary material for: Contrasting parental roles shape sex differences in poison frog space use but not navigational performance
Source: eLife. 2022 Nov 15;11:e80483. doi: 10.7554/eLife.80483 (PMC9665844; doi:10.7554/eLife.80483)
Supplement: Supplementary file 1. [file elife-80483-supp1.docx]

**Section A: supplementary statistic tables**

**Supplementary file 1a. Movement extent model summaries.**

|  | *A. femoralis*  log_e_ (movement extent) | | *D. tinctorius*  log_e_ (movement extent) | | *O. sylvatica*  log_e_ (movement extent) | |
| --- | --- | --- | --- | --- | --- | --- |
| *Predictors* | *Estimates (CI)* | *p* | *Estimates (CI)* | *p* | *Estimates (CI)* | *p* |
| (Intercept) | 2.9 (0.65 – 5.2) | **0.014** | 5.9 (4.7 – 7.2) | **<0.001** | 3.4 (2.50 – 4.35) | **<0.001** |
| Sex [male] | 1.0 (0.2 – 1.8) | **0.019** | 0.6 (-0.45 – 1.7) | 0.240 | -0.8 (-1.3 – -0.4) | **0.001** |
| Tracking  duration | 0.1 (-0.03 – 0.3) | 0.101 | -0.02 (-0.1 – 0.1) | 0.564 | 0.01 (-0.1 – 0.1) | 0.831 |
| Observations | 25 | | 19 | | 29 | |
| R^2^ / R^2^ adjusted | 0.34 / 0.28 | | 0.085 / -0.03 | | 0.38 / 0.33 | |

Statistical summary of three linear models with log_e_-transformed movement extent area in *A. femoralis*, *D. tinctorius*, and *O. sylvatica* as the response variable, sex as the predictor, and tracking duration (days) as a covariate. Statistical significance with p < 0.05 is highlighted in bold.

**Supplementary file 1b. Explored area after 50-m translocation model summaries.**

|  | *A. femoralis* 50-m  log_e_ (explored area) | | *D. tinctorius* 50-m  log_e_ (explored area) | | *O. sylvatica* 50-m  log_e_ (explored area) | |
| --- | --- | --- | --- | --- | --- | --- |
| *Predictors* | *Estimates* | *p* | *Estimates* | *p* | *Estimates* | *p* |
| (Intercept) | 17.5 (10.1 – 24.9) | **<0.001** | 5.7 (-0.5 – 11.8) | 0.068 | -19.5 (-84.4 – 45.5) | 0.530 |
| Sex [male] | 0.6 (0.3 – 0.9) | **<0.001** | 0.1 (-0.3 – 0.5) | 0.681 | 0.2 (-0.4 – 0.8) | 0.514 |
| Temp. | -0.5 (-0.8 – -0.3) | **<0.001** | 0.03 (-0.2 – 0.3) | 0.835 | 1.0 (-1.8 – 3.8) | 0.459 |
| Weight | 0.7 (-0.1 – 1.5) | 0.084 | 0.01 (-0.2 – 0.2) | 0.954 | 1.3 (-0.3 – 2.9) | 0.109 |
| Observations | 32 | | 31 | | 18 | |
| R^2^ / R^2^ adjusted | 0.59 / 0.55 | | 0.015 / -0.09 | | 0.23 / 0.06 | |

Statistical summary of three linear models with log_e_-transformed explored area in *A. femoralis*, *D. tinctorius*, and *O. sylvatica* as the response variable, sex as the predictor, and ambient daytime temperature (Temp.) and frog weight as covariates. Statistical significance with p < 0.05 is highlighted in bold.

**Supplementary file 1c. Homing duration model summaries.**

|  | *A. femoralis* 50-m  log_e_ (homing duration) | | *D. tinctorius* 50-m  log_e_ (homing duration) | | *D. tinctorius* 200-m  log_e_ (homing duration) | | *O.* *sylvatica* 50-m  log_e_ (homing duration) | |
| --- | --- | --- | --- | --- | --- | --- | --- | --- |
| *Predictors* | *Estimates* | *p* | *Estimates* | *p* | *Estimates* | *p* | *Estimates* | *p* |
| (Intercept) | -23.0 (-47.2 – 1.2) | 0.061 | 2.3 (-15.95 – 20.5) | 0.801 | -2.8 (-19.7 – 14.1) | 0.720 | -70.8 (-140.2 – -1.45) | **0.046** |
| Sex [male] | -2.0 (-3.15 – -0.9) | **0.002** | -0.7 (-1.95 – 0.5) | 0.218 | -1.55 (-4.1 – 1.0) | 0.202 | 0.4 (-0.4 – 1.1) | 0.294 |
| Temp. | 1.2 (0.2 – 2.3) | **0.021** | 0.1 (-0.6 – 0.8) | 0.795 | 0.4 (-0.2 – 1.0) | 0.203 | 3.15 (0.15 – 6.1) | **0.042** |
| Weight | -1.7 (-4.3 – 0.85) | 0.175 | -0.6 (-1.2 – 0.05) | 0.069 | -0.5 (-1.8 – 0.7) | 0.364 | 0.2 (-2.05 – 2.4) | 0.860 |
| Observations | 19 | | 30 | | 14 | | 14 | |
| R^2^ / R^2^ adjusted | 0.54 / 0.44 | | 0.13 / 0.03 | | 0.31 / 0.10 | | 0.385 / 0.20 | |

Statistical summary of four linear models with log_e_-transformed homing duration in *A. femoralis*, *D. tinctorius*, and *O. sylvatica* as the response variable, sex as the predictor, and ambient daytime temperature (Temp.) and frog weight as covariates. Statistical significance with p < 0.05 is highlighted in bold.

**Supplementary file 1d. Sex difference in androgen levels.**

|  | *A. femoralis*  log_e_ (androgens) | | *D. tinctorius*  log_e_ (androgens) | | *O. sylvatica*  log_e_ (androgens) | |
| --- | --- | --- | --- | --- | --- | --- |
| *Predictors* | *Estimates* | *p* | *Estimates* | *p* | *Estimates* | *p* |
| (Intercept) | 2.42 (2.16 – 2.68) | **<0.001** | 3.16 (2.58 – 3.74) | **<0.001** | 2.26 (2.01 – 2.52) | **<0.001** |
| Sex [m] | 0.40 (0.09 – 0.72) | **0.012** | 1.04 (0.33 – 1.75) | **0.004** | 0.41 (0.08 – 0.74) | **0.015** |
| Time point [back home] | 0.12 (-0.19 – 0.44) | 0.444 | -0.16 (-0.71 – 0.38) | 0.556 | 0.07 (-0.21 – 0.35) | 0.631 |
| **Random Effects** | | | | | | |
| σ^2^ | 0.42 | | 0.95 | | 0.24 | |
| τ_00_ | 0.00 _id_ | | 0.52 _id_ | | 0.08 _id_ | |
| ICC |  | | 0.35 | | 0.25 | |
| N | 35 _id_ | | 35 _id_ | | 34 _id_ | |
| Observations | 66 | | 56 | | 51 | |
| Marginal R^2^ / Conditional R^2^ | 0.095 / NA | | 0.157 / 0.455 | | 0.125 / 0.342 | |

Statistical summary of three linear mixed models with log_e_-transformed androgen levels in *A. femoralis*, *D. tinctorius*, and *O. sylvatica* as the response variable, sex and sampling point as the predictors, frog identity as the random factor. Statistical significance with p < 0.05 is highlighted in bold.

**Supplementary file 1e. Androgen influence on spatial variables in *A. femoralis****.*

|  | *A. femoralis*  log_e_ (explored area) | | *A. femoralis*  log_e_ (explored area) | | *A. femoralis*  log_e_ (explored area) | |
| --- | --- | --- | --- | --- | --- | --- |
| *Predictors* | *Estimates* | *p* | *Estimates* | *p* | *Estimates* | *p* |
| (Intercept) | 5.8 (5.4 – 6.1) | **<0.001** | 0.04 (0.02 – 0.1) | **0.014** | 0.9 (0.8 – 0.95) | **<0.001** |
| Androgens | 0.04 (-0.2 – 0.2) | 0.725 | 0.00 (-0.02 – 0.02) | 0.804 | 0.02 (-0.02 – 0.1) | 0.343 |
| Sex [male] | 0.3 (-0.2 – 0.8) | 0.261 | NA |  | NA |  |
| Translocation dist.  [50m] | -0.7 (-1.1 – -0.3) | **0.002** | 0.03 (-0.02 – 0.01) | 0.259 | -0.1 (-0.2 – -0.03) | **0.029** |
| Homing success  [1] | 1.2 (0.7 – 1.8) | **<0.001** | NA |  | NA |  |
| Temp. | NA |  | -0.02 (-0.03 – -0.00) | 0.057 | -0.1 (-0.1 – -0.02) | **0.011** |
| Observations | 35 | | 12 | | 12 | |
| R^2^ / R^2^ adjusted | 0.67 / 0.62 | | 0.705 | | 0.61 | |

Statistical summary of three linear models with log-transformed explored area, homing duration, and homing trajectory straightness in *A. femoralis* as the response variables, androgen levels and sex as the predictors, translocation distance, homing success, and average daytime temperature (Temp.) as covariates. Statistical significance with p < 0.05 is highlighted in bold.

**Supplementary file 1f. Androgen influence on spatial variables in *D. tinctorius.***

|  | ***D. tinctorius***  **Log explored area** | | ***D. tinctorius***  **homing duration** | | ***D. tinctorius***  **trajectory straightness** | |
| --- | --- | --- | --- | --- | --- | --- |
| *Predictors* | *Estimates* | *p* | *Estimates* | *p* | *Estimates* | *p* |
| (Intercept) | 7.24 (6.64 – 7.84) | **<0.001** | 0.02 (-0.02 – 0.09) | 0.462 | 0.52 (0.27 – 0.90) | **0.009** |
| Androgens | 0.33 (0.04 – 0.61) | **0.027** | 0.00 (-0.04 – 0.06) | 0.898 | -0.07 (-0.20 – 0.06) | 0.310 |
| Sex [male] | 0.92 (0.04 – 1.80) | **0.042** | 0.01 (-0.06 – 0.09) | 0.769 | -0.04 (-0.30 – 0.21) | 0.761 |
| Translocation dist.  [50m] | -1.62 (-2.44 – -0.80) | **0.001** | 0.06 (-0.01 – 0.12) | 0.092 | 0.17 (-0.21 – 0.46) | 0.307 |
| Homing success  [1] | 0.51 (-0.34 – 1.36) | 0.222 | NA |  | NA |  |
| Weight | 0.60 (0.18 – 1.02) | **0.008** | NA |  | NA |  |
| Observations | 23 | | 16 | | 12 | |
| R^2^ / R^2^ adjusted | 0.762 / 0.691 | | 0.308 | | 0.207 | |

Statistical summary of three linear models with log_e_-transformed transformed explored area, homing duration, and homing trajectory straightness in *D. tinctorius* as the response variables, androgen levels and sex as the predictors, translocation distance, homing success, and frog weight as covariates. Statistical significance with p < 0.05 is highlighted in bold.

**Supplementary file 1g. Androgen influence on spatial variables in *O. sylvatica.***

|  | *O. sylvatica*  Log explored area | | *O. sylvatica*  homing duration | | *O. sylvatica*  trajectory straightness | |
| --- | --- | --- | --- | --- | --- | --- |
| *Predictors* | *Estimates* | *p* | *Estimates* | *p* | *Estimates* | *p* |
| (Intercept) | 6.40 (6.00 – 6.81) | **<0.001** | 0.05 (0.03 – 0.07) | **0.001** | 0.70 (0.54 – 0.91) | **<0.001** |
| Androgens | -0.06 (-0.31 – 0.18) | 0.589 | 0.01 (-0.00 – 0.02) | 0.188 | 0.21 (0.09 – 0.32) | **0.003** |
| Sex [male] | 0.23 (-0.25 – 0.71) | 0.333 | -0.01 (-0.03 – 0.02) | 0.549 | -0.13 (-0.35 – 0.09) | 0.219 |
| Translocation dist.  [50m] | -0.99 (-1.62 – -0.37) | **0.003** | NA |  | NA |  |
| Homing success  [1] | 1.07 (0.47 – 1.67) | **0.001** | NA |  | NA |  |
| Observations | 28 | | 13 | | 13 | |
| R^2^ / R^2^ adjusted | 0.433 / 0.334 | | 0.113 | | 0.451 | |

Statistical summary of three linear models with log_e_-transformed transformed explored area, homing duration, and homing trajectory straightness in *O. sylvatica* as the response variables, androgen levels and sex as the predictors, translocation distance and homing success as covariates. Statistical significance with p < 0.05 is highlighted in bold.

**Supplementary file 1h. Exploration influence on delta androgen levels.**

|  | *A. femoralis*  delta androgens | | *D. tinctorius*  delta androgens | | *O. sylvatica*  delta androgens | |
| --- | --- | --- | --- | --- | --- | --- |
| *Predictors* | *Estimates* | *p* | *Estimates* | *p* | *Estimates* | *p* |
| (Intercept) | 7.84 (-2.75 – 18.44) | 0.140 | -47.48 (-382.53 – 287.57) | 0.768 | 2.64 (-12.97 – 18.25) | 0.717 |
| Explored area | 12.70 (3.37 – 22.03) | **0.010** | 12.77 (-107.77 – 133.31) | 0.825 | 0.53 (-7.61 – 8.67) | 0.889 |
| Sex [male] | 12.70 (-3.73 – 29.13) | 0.124 | 41.74 (-144.22 – 227.70) | 0.641 | -6.99 (-21.00 – 7.03) | 0.296 |
| Tracking  duration | -7.18 (-14.87 – 0.52) | 0.066 | 4.14 (-187.48 – 195.76) | 0.964 | 4.01 (-10.97 – 18.99) | 0.567 |
| Homing success  [1] | -28.63 (-53.37 – -3.88) | **0.025** | 39.40 (-449.42 – 528.21) | 0.866 | 6.24 (-22.51 – 34.99) | 0.642 |
| Observations | 31 | | 21 | | 16 | |
| R^2^ / R^2^ adjusted | 0.353 / 0.254 | | 0.027 / -0.216 | | 0.141 / -0.171 | |

Statistical summary of three linear models with delta androgen levels in *A. femoralis*, *D. tinctorius*, and *O. sylvatica* as the response variable, explored area and sex as the predictors, tracking duration and homing success as covariates. Statistical significance with p < 0.05 is highlighted in bold.

**Section B: supplementary method tables**

**Supplementary file 1i. Study site characteristics.**

| **Site name** | **Locality** | **Coordinates** | **Area (ha)** | **Site characteristic** | **Breeding sites** |
| --- | --- | --- | --- | --- | --- |
| Nouragues island | Nouragues Nature Reserve,  French Guiana | 4°02' N, 52°41' W | 4.6 | Free-range experimental island population | Mostly artificial |
| Nouragues main | Nouragues Nature Reserve,  French Guiana | 4°02' N, 52°41' W | 25 | Natural population | Mostly natural |
| Florida | La Florida, Ecuador | 0°15' S, 79°02' W | 0.5 | Free-range experimental enclosure population | Natural and artificial |
| Canandé | Reserva Canandé, Ecuador | 0°32' N, 79°13' W | 5.5 | Natural population | Natural |

**Supplementary file 1j. Data type and dataset descriptive.**

| **Data types** | **Dataset id** | **Study period** | **Species** | **Site** | **N frogs tagged** | **N frogs included** | **Note** |
| --- | --- | --- | --- | --- | --- | --- | --- |
| Space use tracking | af16 | 2016/02/08 - 2016/03/20 | *A. femoralis* | Nouragues island | 24 | 17 | Only females tracked |
|  | af18 | 2018/03/17 - 2018/04/18 | *A. femoralis* | Nouragues island | 12 | 12 | Only males tracked |
|  | dt16 | 2016/01/27 - 2016/03/19 | *D. tinctorius* | Nouragues main | 31 | 26 |  |
|  | os17 | 2017/04/23 - 2017/05/16 | *O. sylvatica* | Florida | 31 | 29 |  |
| Space use recapture | dt_recap | 2009/01/09 - 2011/06/05 | *D. tinctorius* | Nouragues main | *NA* | 154 | Long-term  recapture data |
|  | af_recap | 2014/01/24 - 2019/04/22 | *A. femoralis* | Nouragues main | *NA* | 165 | Long-term  recapture data |
| Navigation | af17 | 2017/01/27 - 2017/03/11 | *A. femoralis* | Nouragues main | 34 | 28 |  |
|  | dt17 | 2017/02/26 - 2017/03/23 | *D. tinctorius* | Nouragues main | 36 | 29 |  |
| Navigation, androgens, space use tracking | os19 | 2019/05/21 - 2019/06/25 | *O. sylvatica* | Canandé | 52 | 39 | Dataset also used for space use validation |
| Navigation, androgens | dt19 | 2019/02/19 - 2019/03/26 | *D. tinctorius* | Nouragues main | 47 | 38 |  |
|  | af20 | 2020/01/21 - 2020/03/16 | *A. femoralis* | Nouragues main | 44 | 36 |  |

**Supplementary file 1k. Dependent variables definitions used in the statistical analyses.**

| **Data types** | **Variable name** | **Definition** | **Datasets** |
| --- | --- | --- | --- |
| Space use | home range | Area under the 95% utilization density contour | af16, af18, dt16, os17 (tracked > 7 days) |
|  | extent area | Area under the minimum convex polygon | af16, af18, dt16, os17 (tracked > 7 days) |
|  | daily travel | Cumulative daily distance traveled | af16, af18, dt16, os17, os19 (tracked > 2 days) |
|  | extent distance | Maximum linear distance between locations of one individual | af16, af18, dt16, os17, os19 (tracked > 2 days)  dt_recap, af_recap (recaptured >30 days) |
| Navigation androgens | homing success | Homing (yes/no) at least 70% of translocation distance | af17, dt17, os19, dt19, af20  (all translocated frogs) |
|  | explored area | Area within five meters of the movement trajectory | af17, dt17, os19, dt19, af20  (all translocated frogs) |
|  | trajectory straightness | Ratio between straight-line and cumulative distance from the release site to the end of the homing trajectory | af17, dt17, os19, dt19, af20  (successfully homing frogs) |
|  | homing duration | Daytime hours from the release time to the arrival within 10-meters of the home area polygon | af17, dt17, os19, dt19, af20  (homing frogs) |
| Navigation | angular deviation | Angular deviation from home center direction measured at ~20% of the translocation distance from the release site | af17, dt17, os19, dt19, af20  (all translocated frogs) |
| Androgens | androgen level | Water-borne androgen concentration measured within 2 day before translocation and after returning home | os19, dt19, af20  (all translocated frogs) |
|  | delta androgens | Difference between androgen concentrations measured back at the home site and baseline androgens. | os19, dt19, af20  (all translocated frogs) |

**Supplementary file 1l. Predictor variable definitions use in the statistical analyses.**

| **Predictor** | **Definition** |
| --- | --- |
| Species | Three level factor: *A. femoralis, D. tinctorius, O. sylvatica* |
| Sex | Two level factor: male, female |
| Behavior | Three or two level factor: parental, mating, other |
| Weight | Continuous numeric: frog weight in grams |
| Daytime temperature | Continuous numeric: average daytime temperature measured between sunrise and sunset |
| Mean temperature | Continuous numeric: daytime temperature averaged over the entire tracking period of an individual |
| Translocation distance | Two level factor: 50-m, 200-m |
| Homing success | Two level factor: yes, no |
| Explored area | Continuous numeric: area within five meters of the movement trajectory |
| Tracking duration | Continuous numeric: total tracking duration of an individual |
| Period | Count numeric: Number of days between recaptures of the same individual |
| Baseline androgens | Continuous numeric: Water-borne androgen concentration measured within 2 day before translocation |
| Time point | Two level factor: baseline, back home |

**Supplementary file 1m. All models performed for the statistical analyses.**

| **Data type** | **Dependent variable** | **Predictors** | **Model id** | **Datasets** | **Data subset** |
| --- | --- | --- | --- | --- | --- |
| Space use | Log_e_  (home range) | species, sex, tracking duration | m11_allud | af16 + af18 + dt16 + os17 | All species |
|  |  | sex, tracking duration | m12_afud | af16 + af18 | *A. femoralis* |
|  |  | sex, tracking duration | m13_dtud | dt16 | *D. tinctorius* |
|  |  | sex, tracking duration | m14_osud | os17 | *O. sylvatica* |
|  | Log_e_  (extent area) | species, sex, tracking duration | m15_allmcp | af16 + af18 + dt16 + os17 | All species |
|  |  | sex, tracking duration | m16_afmcp | af16 + af18 | *A. femoralis* |
|  |  | sex, tracking duration | m17_dtmcp | dt16 | *D. tinctorius* |
|  |  | sex, tracking duration | m18_osmcp | os17 | *O. sylvatica* |
|  | Log_e_  (daytime travel) | sex, species, points per day | m1_alldl | af16 + af18 + dt16 + os17 | All species |
|  |  | sex, behavior, daytime temperature,  points per day^1^ | m2_afdl | af16 + af18 | *A. femoralis* |
|  |  | behavior, daytime temperature | m4_affdl | af16 | *A. femoralis* females |
|  |  | behavior, daytime temperature | m3_afmdl | af18 | *A. femoralis* males |
|  |  | sex, behavior, daytime temperature,  points per day^1^ | m5_dtdl | dt16 | *D. tinctorius* |
|  |  | behavior^2^ | m7_dtfl | dt16 | *D. tinctorius* females |
|  |  | behavior^2^ | m6_dtml | dt16 | *D. tinctorius* males |
|  |  | sex, behavior, daytime temperature,  points per day^1^ | m8_osdl | os17 | *O. sylvatica* |
|  |  | behavior^2^ | m10_osmdl | os17 | *O. sylvatica* females |
|  |  | behavior^2^ | m9_osfdl | os17 | *O. sylvatica* males |
| Space use validation | Log_e_  (extent distance) | sex, tracking duration | m19_aftr | af16 + af18 | *A. femoralis* tracking |
|  |  | sex, period | m20_afcr | af_recap | *A. femoralis* recapture |
|  |  | sex, tracking duration | m21_dttr | dt16 | *D. tinctorius* tracking |
|  |  | sex, period | m22_dtcr | dt_recap | *D. tinctorius* recapture |
|  |  | sex, tracking duration | m23_oscan | os19 | *O. sylvatica* tracking natural site |
|  |  | sex, tracking duration | m24_osoto | os17 | *O. sylvatica* tracking enclosures |
|  | Log_e_  (daytime travel) | sex | m25_oscan | os19 | *O. sylvatica* tracking natural site |
|  |  | sex | m26_osoto | os17 | *O. sylvatica* tracking enclosures |
| Navigation | Homing success | sex, weight, mean temperature | m1_afprob | af17 + af20 | *A. femoralis* 50-m |
|  |  | sex, weight, mean temperature | m2_dtprob | dt17 + dt19 | *D. tinctorius* 200-m |
|  |  | sex, mean temperature^3^ | m3_osprob | os19 | *O. sylvatica* 50-m |
|  | Log_e_  (explored area) | sex, weight, mean temperature | m4_af50ex | af17 + af20 | *A. femoralis* 50-m |
|  |  | sex, weight, mean temperature | m5_af200ex | af17 + af20 | *A. femoralis* 200-m |
|  |  | sex, weight, mean temperature | m6_dt50ex | dt17 + dt19 | *D. tinctorius* 50-m |
|  |  | sex, weight, mean temperature | m7_dt200ex | dt17 + dt19 | *D. tinctorius* 200-m |
|  |  | sex, weight, mean temperature | m8_os50ex | os19 | *O. sylvatica* 50-m |
|  |  | sex, weight, mean temperature | m9_os200ex | os19 | *O. sylvatica* 200-m |
|  | Trajectory straightness | sex, mean temperature | m10_af50sc | af17 + af20 | *A. femoralis* 50-m homing |
|  |  | sex, mean temperature | m11_dt50sc | dt17 + dt19 | *D. tinctorius* 50-m homing |
|  |  | sex, mean temperature | m12_dt200sc | dt17 + dt19 | *D. tinctorius* 200-m homing |
|  |  | sex, mean temperature | m13_os50sc | os19 | *O. sylvatica* 50-m homing |
|  | Log_e_  (homing duration) | sex, weight, mean temperature | m14_af50dr | af17 + af20 | *A. femoralis* 50-m homing |
|  |  | sex, weight, mean temperature | m15_dt50dr | dt17 + dt19 | *D. tinctorius* 50-m homing |
|  |  | sex, weight, mean temperature | m16_dt200dr | dt17 + dt19 | *D. tinctorius* 200-m homing |
|  |  | sex, weight, mean temperature | m17_os50dr | os19 | *O. sylvatica* 50-m homing |
|  | Angular deviation | sex | m18_af50cr | af17 + af20 | *A. femoralis* 50-m |
|  |  | sex | m19_dt50cr | dt17 + dt19 | *D. tinctorius* 50-m |
|  |  | sex | m20_dt200cr | dt17 + dt19 | *D. tinctorius* 200-m |
|  |  | sex | m21_os50cr | os19 | *O. sylvatica* 50-m |
| Androgens | Log_e_  (baseline androgens) | sex, time point | m1_afT | af20 | *A. femoralis* |
|  |  | sex, time point | m1_dtT | dt19 | *D. tinctorius* |
|  |  | sex, time point | m1_osT | os19 | *O. sylvatica* |
|  | Homing success | baseline androgens, sex,  translocation distance^4^ | m2_afT | af20 | *A. femoralis* |
|  |  | baseline androgens, sex,  translocation distance^4^ | m2_dtT | dt19 | *D. tinctorius* |
|  |  | baseline androgens, sex, translocation distance, weight^4^ | m1_osT | os19 | *O. sylvatica* |
|  | Log_e_  (explored area) | baseline androgens, sex, translocation distance, homing success^4^ | m3_afT | af20 | *A. femoralis* |
|  |  | baseline androgens, sex, translocation distance, homing success, weight^4^ | m3_dtT | dt19 | *D. tinctorius* |
|  |  | baseline androgens, sex, translocation distance, homing success^4^ | m3_osT | os19 | *O. sylvatica* |
|  | Homing duration | baseline androgens, translocation distance, mean temperature^4^ | m4_afT | af20 | *A. femoralis* homing |
|  |  | baseline androgens, sex, translocation distance^4^ | m4_dtT | dt19 | *D. tinctorius* homing |
|  |  | baseline androgens, sex^4^ | m4_osT | os19 | *O. sylvatica* homing |
|  | Trajectory straightness | baseline androgens, translocation distance, mean temperature^4^ | m5_afT | af20 | *A. femoralis* homing |
|  |  | baseline androgens, sex, translocation distance^4^ | m5_dtT | dt19 | *D. tinctorius* homing |
|  |  | baseline androgens, sex^4^ | m5_osT | os19 | *O. sylvatica* homing |
|  | Delta androgens | explored area, sex, tracking duration, homing success^4^ | m6_afT | af20 | *A. femoralis* homing |
|  |  | explored area, sex, tracking duration, homing success^4^ | m6_dtT | dt19 | *D. tinctorius* homing |
|  |  | explored area, sex, tracking duration, homing success^4^ | m6_osT | os19 | *O. sylvatica* homing |

^1^ Full model.

^2^ Daytime temperature excluded based on model selection for both sexes.

^3^ Weight excluded because the model with weight didn’t converge.

^4^ Weight and/or mean temperature were excluded to reduce the number of predictors if the model excluding these predictors (separately) was not significantly different (P > 0.1) from the full model.
